# Diagnostic accuracy of “sweeping” method compared to conventional sampling in rapid urease test for *Helicobacter pylori* detection in atrophic mucosa

**DOI:** 10.1038/s41598-020-75528-1

**Published:** 2020-10-28

**Authors:** Choong-Kyun Noh, Gil Ho Lee, Jin Woong Park, Jin Roh, Jae Ho Han, Eunyoung Lee, Bumhee Park, Sun Gyo Lim, Sung Jae Shin, Jae Youn Cheong, Jin Hong Kim, Kee Myung Lee

**Affiliations:** 1 Department of Gastroenterology, Ajou University School of Medicine, 164, World Cup-ro, Yeongtong-gu, Suwon, 16499 Republic of Korea; 2 Department of Pathology, Ajou University School of Medicine, Suwon, Republic of Korea; 3 Office of Biostatistics, Ajou Research Institute for Innovation Medicine, Ajou University Medical Center, Suwon, Republic of Korea

**Keywords:** Oesophagogastroscopy, Gastrointestinal diseases

## Abstract

Although the rapid urease test (RUT) is a simple method for detecting *Helicobacter pylori* (*H. pylori*) infection, it requires sufficient biopsy samples and its sensitivity varies depending on the site and condition of *H. pylori* infection. We compared the diagnostic performance of a “sweeping method” for *H. pylori* detection with the conventional biopsy sampling method in atrophic gastric conditions which can reduce RUT accuracy. This prospective study included 279 patients who underwent upper endoscopy to determine the presence of *H. pylori* infection. Gastric mucosa of both the antrum and the corpus were swabbed, and we named this method the “sweeping method”. Biopsy sampling for the conventional method, histologic evaluation, and polymerase chain reaction were performed at the same time. The sensitivity, specificity, and accuracy of the sweeping method were 0.941, 0.826, and 0.903, respectively, compared to 0.685, 0.859, and 0.742, respectively, for the conventional biopsy method. The area under the receiver operating curve for the sweeping method was 0.884 versus 0.772 for the conventional method (*P* < 0.001). The sweeping method had a faster detection time than the conventional method. Compared to conventional biopsy sampling, the sweeping method with the RUT provided higher sensitivity and accuracy for the detection of *H. pylori*, with a faster detection time.

## Introduction

Although the rate of *Helicobacter pylori* (*H. pylori*) infection has been decreasing, its prevalence is still high worldwide^1^. *H. pylori* infection is associated with various diseases, including gastritis, peptic ulcer, gastric cancer, and mucosa-associated lymphoid tissue lymphoma^2,3^. A meta-analysis provided evidence of an association between *H. pylori* eradication and a reduction in the incidence of gastric cancer^4^. Moreover, compared with a placebo treatment, *H. pylori* eradication has been shown to decrease the rate of metachronous gastric cancer after endoscopic resection of early gastric cancer^5,6^. Thus, accurate detection of *H. pylori* infection is crucial.

The rapid urease test (RUT) is the commonly used invasive method for *H. pylori* detection, where tissue samples from the gastric mucosa are placed into a commercially available analysis kit, with results, interpreted from a change in color, requiring minutes to hours. The tissue sample must be obtained from a site where the organisms are present, and a sufficient amount of *H. pylori* organisms must be included in the sample to obtain accurate results^7,8^. Moreover, the use of antibiotics, bismuth-containing compounds, and proton-pump inhibitor (PPI) may yield false-negatives as the bacterial density is reduced^7,9,10^. The detection rate of these biopsy-based tests is further reduced by gastric atrophy, intestinal metaplasia, and peptic ulcer bleeding^11–14^. The collection of multiple biopsies from a site has been proposed to increase the sensitivity of the RUT^14,15^, but does increase the risk of mucosal damage and adverse events, such as bleeding.

*Helicobacter pylori* detection using the RUT is possible, foremost, because the organism is present in the mucus layer covering the mucosa^16^. Since *H. pylori* is a non-invasive bacterium living in the mucus layer, the essential component for the urease test is the mucus itself, with the mucosal and submucosal issues actually not needed for diagnosis. For this reason, the RUT does not directly detect *H. pylori*; rather, RUT-based detection of *H. pylori* is indirect, via the detection of urease which is expressed by the organism^8^.

We attempted to develop a method for *H. pylori* detection that would address the drawbacks of conventional biopsy-based RUT. Our “sweeping method” consists of collecting as many *H. pylori* organisms as possible by absorbing the gastric mucus using swabs, without requiring direct sampling of the gastric tissue. We hypothesized that this sweeping method could provide a more accurate and faster detection of *H. pylori* infection than the conventional biopsy sampling method with the RUT, with better safety profile in lowering the risk for adverse events. Thus, this prospective study aimed to evaluate the diagnostic performance of our sweeping method against the conventional biopsy sampling method, for the detection of *H. pylori* infection using RUT.

## Results

### Baseline characteristics

A total of 279 patients were prospectively enrolled into the study, and the four *H. pylori* tests were performed in all patients. Baseline patient characteristics are summarized in Table 1. The mean age was 59.76 years, and 69.2% were men. Of the 279 patients, 243 (87.1%) had an ulcer, cancer, mucosa-associated lymphoid tissue lymphoma, or adenoma. Overall, atrophy or metaplasia was confirmed by histopathology in 245 (87.8%) patients.

**Table 1:** Baseline characteristics of enrolled patients (n = 279).

Characteristics	Value
**Age, years**	
Mean ± SD	59.76 ± 12.07
**Gender, n (%)**	
Male	192 (69.2)
Female	86 (30.8)
BMI, m^2^/kg (mean ± SD)	24.19 ± 3.14
**Medical history, n (%)**	
Hypertension	106 (38.0)
Diabetes	46 (16.5)
Cerebrovascular accident	11 (3.9)
Cardiovascular disease	5 (1.8)
Liver cirrhosis	3 (1.1)
**Reason of the endoscopy, n (%)**	
Routine check-up	36 (12.9)
Reflux symptoms	6 (2.2)
Melena	5 (1.8)
Adenoma work-up	78 (28.0)
Cancer work-up	108 (38.7)
Ulcer follow-up	36 (12.9)
MALT lymphoma work-up	10 (3.6)
**RUT indication, n (%)**	
Ulcer	47 (16.9)
Adenoma	78 (28.0)
Cancer	150 (53.8)
MALT lymphoma	9 (3.2)
Patient wanted	36 (12.9)
**Endoscopic diagnosis, n (%)**	
Gastric ulcer	39 (14.0)
Duodenal ulcer	8 (2.9)
Gastric adenoma	78 (28.0)
Early gastric cancer	72 (25.8)
Advanced gastric cancer	37 (13.3)
MALT lymphoma	9 (3.2)
Other gastritis	6 (2.2)
Normal	30 (10.8)
*H. pylori* infection in gold standard, n (%)	187 (67.0)
Atrophy without metaplasia, n (%)	63 (22.6)
Atrophy with metaplasia, n (%)	182 (65.2)
Antiplatelet use, n (%)	28 (10.0)
PPI use within 2 weeks, n (%)	50 (17.9)

*SD* standard deviation; *BMI* body mass index; *MALT* mucosa-associated lymphoid tissue; *RUT* rapid urease test; *H. pylori Helicobacter pylori*; *PPI* proton-pump inhibitor.

### *Helicobacter pylori* infection status

Overall, 187 (67.0%) patients tested positive for *H. pylori* infection using the gold standard definition. The remaining 92 (33.0%) patients tested negative for *H. pylori*, of which a negative result was obtained on all four tests in 69 (75.0%) patients. The sweeping method detected not only 176 (63.1%) cases but also 16 (17.4%) of the gold standard-negative cases. *H. pylori-*positive rates were as follows: sweeping method, 68.8%; conventional method, 50.5%; histology, 64.2%; and PCR, 63.1%. The *H. pylori*-positive rate for the sweeping method was significantly higher than that for the conventional, histology, or PCR method (*P* < 0.001). There were 260 patients (93.2%) whose IHC and PCR results matched.

### Diagnostic performance of the sweeping method for* H. pylori *detection

**Figure 1:**
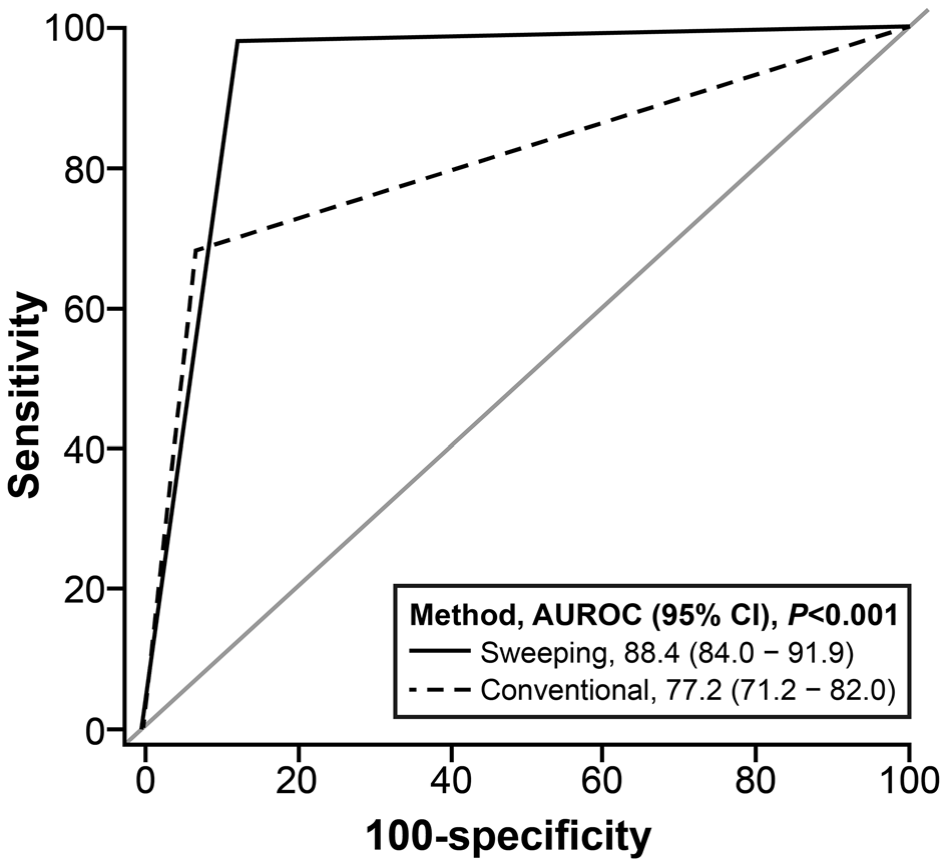
Area under the receiver operating characteristic curve for the sweeping and the conventional method.

The diagnostic performance of the sweeping and conventional methods is shown in Table 2 and Supplementary Fig. 1. The sensitivity of the sweeping method was 0.941 (95% CI, 0.897–0.970), which was higher than that for the conventional method at 0.685 (95% CI, 0.613–0.750). The specificity of the sweeping was 0.826 (95% CI, 0.733–0.897) versus 0.859 (95% CI, 0.771–0.923) for the conventional method. The overall accuracy rate of *H. pylori* detection for the sweeping method was 0. 903 (95% CI, 0.862–0.935) versus 0.742 (95% CI, 0.686–0.792) for the conventional method. Therefore, the sweeping method has higher sensitivity, accuracy, PPV, and NPV than the conventional method for *H. pylori* detection. The AUROC for the sweeping method was 0.884 (95% CI, 0.840–0.919) versus 0.772 (95% CI, 0.718–0.820) for the conventional method (*P* < 0.001) (Fig. 1). In addition, there was a high agreement between the results of the sweeping method and those of IHC staining (overall kappa value, 0.851) and those of PCR (overall kappa value, 0.785) (Table 3). Therefore, the sweeping method demonstrated superior detection capability than the conventional method.

**Table 2:** Diagnostic performance of the sweeping method compared to the conventional method for detection of *Helicobacter pylori* infection.

Performance characteristic (95% CI)	Rapid urease test	
	Sweeping	Conventional
Sensitivity	0.941 (0.897–0.970)	0.685 (0.613–0.750)
Specificity	0.826 (0.733–0.897)	0.859 (0.771–0.923)
Accuracy	0.903 (0.862–0.935)	0.742 (0.686–0.792)
PPV	0.917 (0.868–0.952)	0.908 (0.848–0.950)
NPV	0.874 (0.785–0.935)	0.573 (0.486–0.656)

*CI* confidence interval; *PPV* positive predictive value; *NPV* negative predictive value.

**Table 3:** Diagnostic performance of the sweeping method based on time for detection and agreement with the conventional method, histology examination, and polymerase chain reaction.

Time	Sensitivity (%)	Specificity (%)	Accuracy (%)	PPV (%)	NPV (%)	Kappa value^a^		
						Conventional	Histology	PCR
≤ 5 min (n = 232)	0.971	0.882	0.935	0.925	0.954	0.535	0.835	0.794
≤ 15 min (n = 254)	0.975	0.872	0.937	0.929	0.954	0.492	0.844	0.781
≤ 30 min (n = 268)	0.977	0.872	0.940	0.934	0.954	0.468	0.848	0.780
≤ 60 min (n = 279)	0.978	0.872	0.943	0.938	0.954	0.470	0.851	0.785

*PPV* positive predictive value; *NPV* negative predictive value; *PCR* polymerase chain reaction.

^a^Kappa value < 0.00, poor; 0.00–0.20, slight; 0.21–0.40, fair; 0.41–0.60, moderate; 0.61–0.80, substantial; 0.81–1.00, almost perfect.

### Time to positive results of sweeping versus conventional methods

The mean time to *H. pylori* detection for the sweeping method was 9.12 ± 13.62 min, which was faster than that for the conventional method (14.58 ± 17.48 min, *P* = 0.003). With the sweeping method, results were determined in ≤ 10 min in 155 (80.7%) cases of all *H. pylori-*positive cases, and in ≤ 5 min in 143 (74.5%) cases (Supplementary Fig. 2). The results of the sweeping method were consistent at all time points: ≤ 5, ≤ 15, ≤ 30, and ≤ 60 min (Table 3).

### Performance characteristics for various conditions

We analyzed the diagnostic performance of the sweeping and conventional methods for various conditions: location, atrophy with/without metaplasia, PPI use, ulcer, and cancer (Table 4). In all conditions, the sensitivity and accuracy rates of the sweeping method remained higher than those of the conventional method. Interestingly, the sweeping method still showed high diagnostic performance of both the on-PPI group and off-PPI group. The sweeping method had high sensitivity, specificity, accuracy, PPV, and NPV in patients with ulcers or gastric cancer, for whom accurate diagnosis is important.

**Table 4:** Comparison of the diagnostic performance of the sweeping method and the conventional biopsy sampling method for various conditions (location (antrum/corpus), atrophy with/without metaplasia, use of proton-pump inhibitor, presence of a peptic ulcer, and gastric cancer).

Performance characteristics (95% CI)	Antrum (n = 279)		Corpus (n = 279)	
	Sweeping	Conventional	Sweeping	Conventional
Sensitivity	0.893 (0.840–0.933)	0.551 (0.477–0.624)	0.829 (0.767–0.880)	0.588 (0.514–0.660)
Specificity	0.804 (0.709–0.880)	0.957 (0.892–0.988)	0.848 (0.758–0.914)	0.880 (0.796–0.939)
Accuracy	0.864 (0.818–0.902)	0.685 (0.627–0.739)	0.835 (0.786–0.877)	0.685 (0.627–0.739)
PPV	0.903 (0.851–0.941)	0.963 (0.907–0.990)	0.917 (0.865–0.954)	0.909 (0.843–0.954)
NPV	0.787 (0.691–0.865)	0.512 (0.434–0.589)	0.709 (0.615–0.792)	0.513 (0.432–0.593)

Performance characteristics (95% CI)	Atrophy only (n = 63)		Atrophy with metaplasia (n = 182)	
	Sweeping	Conventional	Sweeping	Conventional
Sensitivity	0.898 (0.778–0.966)	0.714 (0.567–0.834)	0.959 (0.906–0.986)	0.661 (0.570–0.745)
Specificity	0.643 (0.351–0.872)	0.929 (0.661–0.998)	0.820 (0.700–0.906)	0.853 (0.738–0.930)
Accuracy	0.841 (0.727–0.921)	0.762 (0.638–0.860)	0.912 (0.861–0.949)	0.725 (0.654–0.789)
PPV	0.898 (0.778–0.966)	0.972 (0.855–0.999)	0.913 (0.850–0.956)	0.899 (0.817–0.953)
NPV	0.643 (0.351–0.872)	0.482 (0.287–0.681)	0.909 (0.801–0.970)	0.559 (0.452–0.662)

Performance characteristics (95% CI)	On-PPI (n = 50)		Off-PPI (n = 229)	
	Sweeping	Conventional	Sweeping	Conventional
Sensitivity	0.926 (0.757–0.991)	0.643 (0.441–0.814)	0.944 (0.896–0.974)	0.675 (0.597–0.747)
Specificity	0.870 (0.664–0.972)	0.870 (0.664–0.972)	0.812 (0.699–0.896)	0.855 (0.750–0.928)
Accuracy	0.900 (0.782–0.967)	0.800 (0.663–0.900)	0.904 (0.858–0.939)	0.729 (0.667–0.786)
PPV	0.893 (0.718–0.977)	0.870 (0.664–0.972)	0.921 (0.868 –0.957)	0.915 (0.850–0.959)
NPV	0.909 (0.708–0.989)	0.741 (0.537–0.889)	0.862 (0.753–0.935)	0.532 (0.435–0.627)

Performance characteristics (95% CI)	Ulcer (n = 47)		Cancer (n = 109)	
	Sweeping	Conventional	Sweeping	Conventional
Sensitivity	0.926 (0.757–0.991)	0.630 (0.424–0.806)	0.936 (0.857–0.979)	0.667 (0.551–0.769)
Specificity	0.900 (0.683–0.988)	1.000 (0.832–1.000)	0.839 (0.663–0.946)	0.807 (0.625–0.926)
Accuracy	0.915 (0.796–0.976)	0.787 (0.643–0.893)	0.908 (0.838–0.955)	0.706 (0.612–0.790)
PPV	0.926 (0.757–0.991)	1.000 (0.805–1.000)	0.936 (0.857–0.979)	0.897 (0.788–0.961)
NPV	0.900 (0.683–0.988)	0.667 (0.472–0.827)	0.839 (0.663–0.946)	0.490 (0.348–0.634)

*CI* confidence interval; *PPV* positive predictive value; *NPV* negative predictive value; *PPI* proton-pump inhibitor.

### Adverse events

A significant difference was found in the adverse event between the two methods (*P* < 0.001) (Supplementary Table 1). In the conventional method, 29 cases of oozing blood (29/30, 96.7%) were spontaneously controlled without treatment, the remaining one case and two spurting cases were treated using hemostatic coagulation. All coagulation cases occurred in patients who did not stop taking the antiplatelet. In the sweeping method, there were only three cases of oozing blood, and all cases resolved spontaneously. Superficial damage was found in six (2.2%) cases in the sweeping method, while 33 (11.8%) cases of submucosal exposure and 11 (3.9%) cases of muscle exposure were observed in the conventional method, where tissue damage was inevitable because of iatrogenic tissue removal.

## Discussion

Through this prospective study, we developed a new method using a commercially available RUT kit that overcomes the drawbacks of the conventional biopsy sampling method. Our sweeping method provided a high detection rate and rapid detection time for *H. pylori* infection, regardless of the conditions. Moreover, our study confirmed that this method is safe, without concern for bleeding or mucosal damage.

Although bacteria have difficulty thriving in a gastric environment, *H. pylor*i can survive by adapting to gastric conditions. Since *H. pylori* produces urease, which can neutralize gastric acid by generating alkali, *H. pylori* can survive in the mucus layer by swimming or adhering to the epithelial cells via multiple bacterial-surface components^17^. In the conventional method for RUT, endoscopic biopsy forceps are used to collect samples of gastric tissue for analysis. The biopsy specimens generally include both mucosal and submucosal tissues, as well as a limited amount of the mucus that lies on the mucosa. Therefore, compared with the conventional method, the sweeping method can acquire more *H. pylori* as it collects gastric mucus from a larger gastric surface area. The earlier color change seen in the sweeping samples might be explained by a higher amount of bacterial load which correlates to the total urease activity in the sample.

In some previous studies, a brushing method was employed for obtaining samples for *H. pylori* detection^18–23^. These studies confirmed results using smears after brushing^18,19^, shaking the brush in the urea broth^20,21^, or incubating the sample taken in a special buffer^22,23^. None of these studies used commercially available RUT kit, making it impossible to verify the results immediately, and the test process was difficult. The brush cost also makes it challenging to use in the clinical field. Moreover, in these studies, detection time was not evaluated and results were not validated in various conditions. However, our sweeping method uses a commercially available kit, which provides the convenience of inspection, fast detection time, and high accuracy.

Sampling error is a major issue of invasive diagnostic methods of *H. pylori* detection, with studies having reported on the conditions that may increase the sampling error during invasive methods^7,9,11,13,14^. We expect that the sweeping method for the RUT may effectively reduce this problem of false-negative results. Compared to the conventional method, the sweeping method may provide more accurate diagnosis of patients who need *H. pylori* eradication, which may inhibit the progression of gastric adenoma to carcinoma in patients with gastric adenoma^4^, and may reduce the rate of metachronous gastric cancer after endoscopic submucosal dissection^5,6^. Thus, accurate detection of *H. pylori* in these patients is important, and a test with higher sensitivity and accuracy, such as our sweeping technique, would be clinically useful in this regard.

Our study showed a low sensitivity for the conventional method, which can be explained in two ways. First, a biopsy sample was taken from both the antrum and corpus, and each sample was placed into separate kits for subgroup analysis. Moon et al. reported that the overall positivity for *H. pylori* of the combined test was superior to that of separate tests (69.2% vs. 64%, *P* < 0.01)^15^. Second, we found a high proportion of atrophy (87.8%) and metaplasia (65.2%) in the biopsy sites among our patients. Studies have demonstrated that biopsy-based tests show a lower *H. pylori* detection rate when mucosal atrophy or intestinal metaplasia is present^11,13,14^. However, the sweeping method was not greatly affected by such local conditions and, thus, provided a higher sensitivity and accuracy than the conventional method.

Our study has potential limitations. First, this study did not include healthy individuals only. Overestimation is possible because a large proportion of patients had chronic diseases associated with *H. pylori*, including ulcer and cancer. However, the conventional method was also conducted in the same clinical population. Therefore, our findings are significant in groups that need treatment, such as patients with gastric ulcers or cancer. Second, we did not compare the diagnostic performance of the sweeping method with that of ^13^C-urea breath test, stool antigen, and culture, making it difficult to interpret the meaning of false positives of the sweeping method. In our study, we found 16 cases of false positives using the sweeping method. In these false-positive cases, the mean time to color change was 3 min, which was ≤ 5 min in all cases. Since we cannot exclude the possibility of true positives, additional investigation is needed to examine the possibility of a true infection that IHC or PCR could not detect. Third, the sweeping method does not acquire histologic information, which is available in biopsy samples. Moreover, biopsy samples can also be re-cycled for PCR. Fourth, the scoring for adverse events was not performed in a blinded fashion. Blinding in this case, however, may not be important as the sweeping method is fundamentally a safe technique, with little risk of any meaningful damage to the mucosa. Fourth, we did not perform a culture for the cause analysis of the sweeping method’s false-positive results. In addition to *H. pylori* that colonizes the hypochlorhydric stomach, other known urease-producing bacterial species such as *Proteus mirabilis, Citrobacter freundii, Klebsiella pneumonia, Enterobacter cloacae* or *Staphylococcus aureus* could be present^24^. Fifth, we performed sweeping and conventional sampling from the antrum and corpus, respectively. In the conventional sampling method, diagnostic performance may be lower when combined biopsy samples from two areas is used. However, the sweeping method showed superior results under the same condition. Finally, we were unable to determine the amount of *H. pylori* and the level of urease absorbed by the swab during the sweeping method. Additionally, we were not able to determine the proper number of sweeps. It is ideal to collect a large number of *H. pylori* with a small number of sweeps, and further study is necessary to determine the appropriate number of sweeps.

In summary, although the RUT is a common and convenient invasive test, it has low sensitivity to *H. pylori* infection due to sampling error. Our sweeping method showed high sensitivity and accuracy, and a fast detection time, regardless of the sampling site and condition. It is a safe method, without any concern for bleeding and mucosal damage as tissue sampling per se is not required. The mucus obtained was analyzed using a commercially available RUT that is widely used in practice, incurring a low additional cost. We expect that the sweeping method will play a role in providing a simple and accurate diagnosis of *H. pylori* infection.

## Methods

This was a prospective, single-center study, conducted at the Ajou University Medical Center (Suwon, Republic of Korea). The study protocol was approved by our institutional review board (Ajou University Hospital Institutional Review Board, approval no. AJIRB-MED-OBS-18-091). Informed consent for study participation was obtained from all patients. This article adhered to the Standards for Reporting of Diagnostic Accuracy Studies^25^. All methods were carried out in accordance with relevant guidelines and regulations.

### Study population

**Figure 2:**
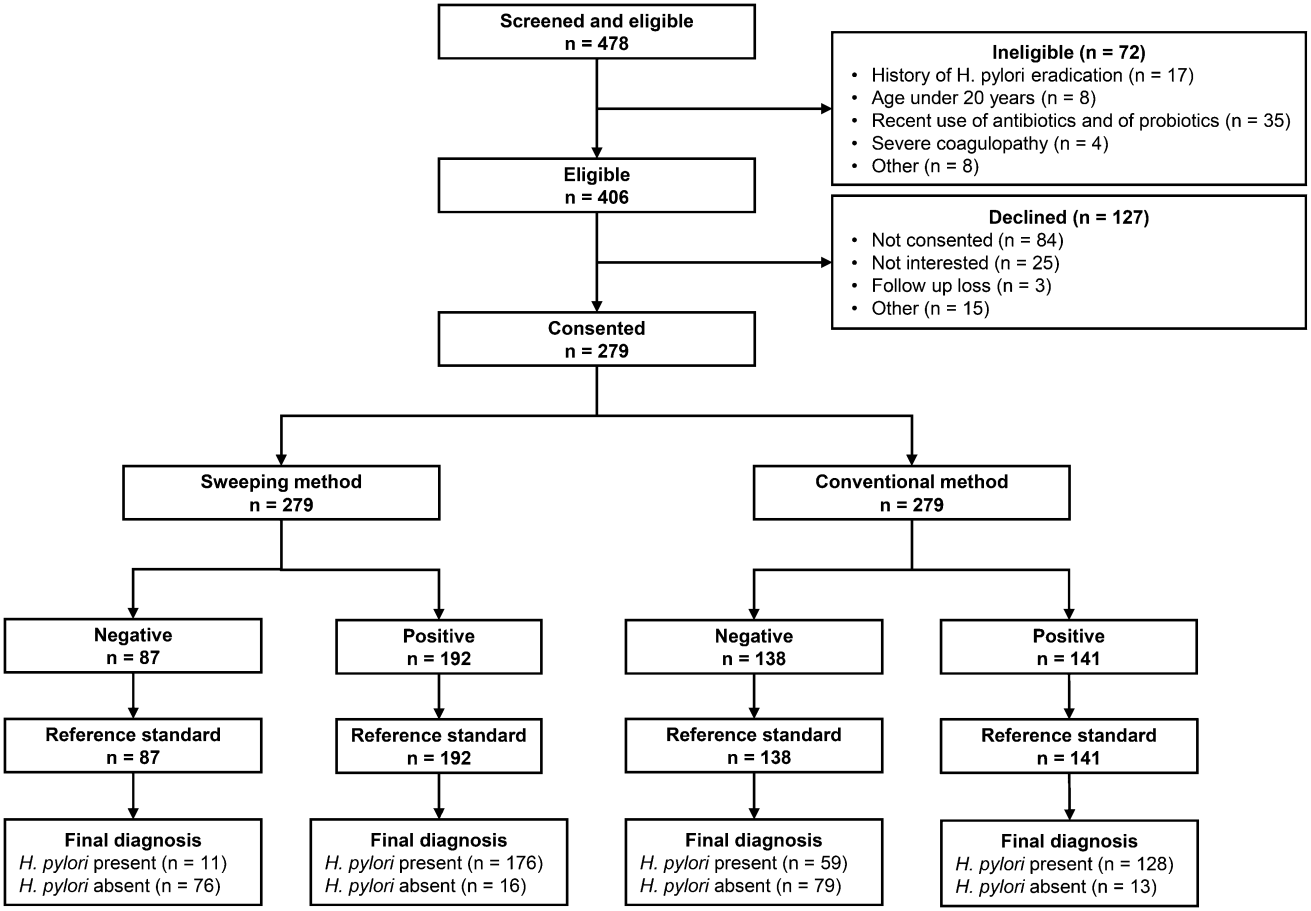
Flow diagram for the selection of the study sample.

Eligible patients were those who underwent upper endoscopy and either required or requested an *H. pylori* test, from November 2018 to July 2019. The exclusion criteria were pregnancy, history of *H. pylori* eradication, age < 20 years, recent use of antibiotics and probiotics (within the last 2 months), contraindication to biopsy due to severe coagulopathy, and unwillingness to participate in the study. General demographic information and details of past medical history, medications (antibiotics, antiplatelet, and ulcer-healing medications including PPI), and reason for a RUT were recorded. Patients who took PPIs within 2 weeks prior to undergoing upper endoscopy were enrolled, and a subgroup analysis was performed for these patients. Figure 2 shows the flow diagram of enrollment of the study participants.

### Endoscopic examination

Upper endoscopy was performed by four expert gastroenterologists, using a one-channel endoscope (Q260J, Olympus Optical Co., Tokyo, Japan). Standard biopsy forceps, with a 6-mm opening diameter, were used in all patients (FB-21K-1, Olympus, Tokyo, Japan). All examinations were done under moderate sedation. In this study, four different methods for the detection of *H. pylori* were used: our novel sweeping method, the conventional biopsy sampling method, histopathological confirmation including immunohistochemical (IHC) staining, and real-time polymerase chain reaction (RT-PCR) using paraffin-embedded tissue. Each method was performed during the same endoscopic procedure, without overlapping of the regions of the gastric mucosa from which the samples were obtained. The order of all methods was randomized (1:1:1 arranged to sweeping, conventional sampling, and histopathology, computer-generated random list). The commercially available *Campylobacter-*like Organism (CLO) kit (PYLO-PLUS, Gulf Coast Scientific, Oldsmar, FL, USA) was used for the RUT. A positive RUT was determined by a change in color from yellow to red within 60 min following sample placement in the kit, at room temperature, as recommended by the manufacturer. The tested kits were kept in a tray, and two experienced nurses determined the color change. For analysis, the color change, using the color matching scheme in the test kit, was evaluated at 1-min intervals for the first 15 min and every 5 min thereafter, up to the 60-min limit. The overall detection time was defined as the shortest time to obtain a result, with the detection time for the antrum and corpus measured separately. Bleeding was assessed as no bleeding, minimal, oozing, and spurting, while damage was assessed as no damage, superficial, submucosal exposure, and muscle exposure (Supplementary Fig. 3).

### Sweeping method

**Figure 3:**
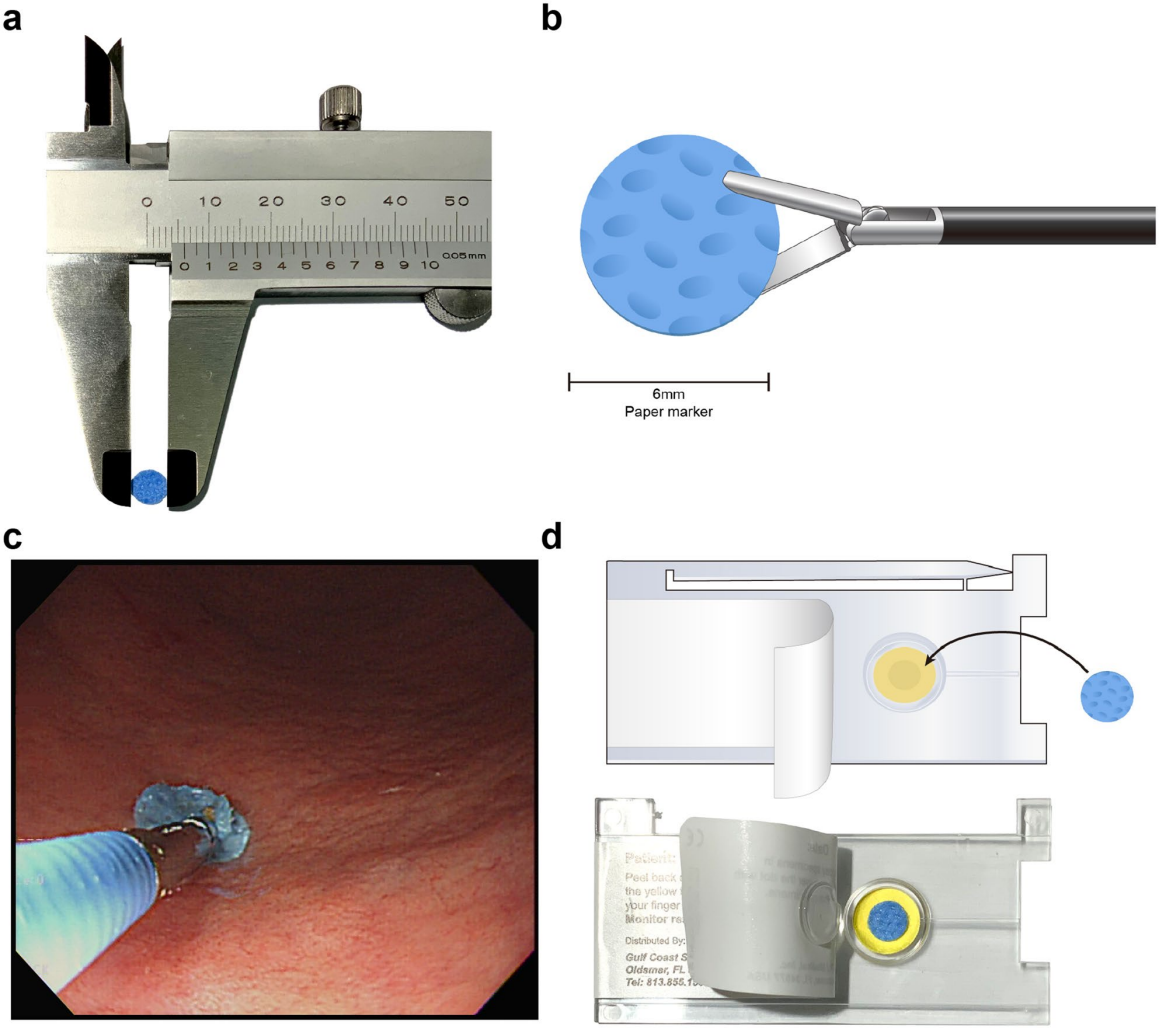
Study design and illustration of the process to perform the sweeping method. The mimetic diagram and an actual image of the absorbent swab is shown (**a**,**b**), for the sweeping method performed in the antrum (**c**). An illustration and an actual image of showing placement of the swab in the detection kit (**d**).

Sweeping is a method of swabbing the mucosa using a sweeping motion with an absorbent swab held with forceps. Sterilized 6-mm circular pieces were punched out from a nonwoven fabric (LIVSEN SMMS BU3, Toray Advanced Materials Korea Inc., Guri, Korea), which is used as surgical or procedure drape (Fig. 3a). Gastric juices were not suctioned during the sweeping. With the forceps grasping the swab (Fig. 3b), the side of the great curvature of the antrum was swept back and forth, 10 times (Fig. 3c, Supplementary Video). Thereafter, the swab was withdrawn from the biopsy channel and placed into the sample insertion well of the CLO kit, and the test result was determined (Fig. 3d). The same sweeping method was repeated for the corpus. Although the endoscopes were disinfected, the possibility of contamination through an endoscope channel was checked before the examination by inserting the swab into the channel to check for the color. The single swab product costs about less than 2 cents.

### Conventional biopsy sampling method

According to the standard protocol for conventional sampling^26^, two tissue samples were collected from the antrum and the corpus. In the antrum, one sample was obtained each in the greater and lesser curvatures, in areas where sweeping was not performed. In the corpus, one sample was obtained 4 cm proximal to the angulus and the second from the middle portion of the greater curvature. Samples were again evaluated using the CLO kit.

### Biopsy and histopathological confirmation

Gastric mucosal biopsies were also obtained from all patients for histopathology examination, two from the antrum (greater and lesser curvature) and two from the corpus (greater and lesser curvature)^27^. All samples were fixed in 10% buffered formalin solution, sent to the pathology department, where they were embedded in paraffin. Hematoxylin and eosin, Giemsa, and IHC staining was performed in all cases to determine the presence of *H. pylori* (Supplementary Fig. 4). Two expert pathologists, who were blinded to the results of the sweeping and conventional biopsy sampling method, performed the analysis.

### Genomic DNA extraction and RT-PCR for detection of *H. pylori* infection

We retrieved the formalin-fixed paraffin-embedded tissue blocks of gastric biopsy specimens from the archives of the pathology department. To obtain sufficient amount of genomic DNA, 7–8 sections of 10 μm thickness were cut from the formalin-fixed paraffin-embedded tissue block. After deparaffinization and rehydration, the DNA extraction procedure was performed with a QIAMP DNA micro kit (Qiagen, Hilden, Germany) according to manufacturer’s instruction. To determine any infection by *H. pylori*, a U-TOP HPY CLAR detection kit (SeaSun Biomaterials, Daejeon, Republic of Korea) was used. RT-PCR was performed using the CFX96 RT-PCR detection system (Bio-Rad, Hercules, CA, USA) according to the manufacturer’s manual. Data were analyzed using BIO-RAD CFX manager v1.6 software (Bio-Rad, Hercules, CA, USA). Presence of *H. pylori* was determined by the fluorescence signal of detection probes and corresponding melting temperature.

### *Helicobacter pylori* infection by gold standard definition

Because both histopathology and PCR have very high sensitivity and specificity^27,28^, we defined *H. pylori* infection status as positive if at least one of the histopathology (IHC) and PCR results was positive. All other cases were considered non-infection.

### Study outcomes

The primary outcome was the diagnostic performance of the sweeping method in detecting *H. pylori* infection*.* The sensitivity, specificity, and accuracy of the sweeping method were analyzed and compared against those of the conventional method. The secondary outcome was the time for detection.

### Statistical analysis

The sample size was calculated to detect a difference of 0.132 between two diagnostic tests whose sensitivities are 0.985 and 0.853, respectively (preliminary study), with a power of 90%. This procedure uses a two-sided McNemar test with a significance level of 0.05. The prevalence of *H. pylori* infection in the general population is 0.530^29^. The proportion of discordant pairs is 0.185. Based on this sample size calculation, a minimum of 200 participants was required. The time to *H. pylori* detection between the conventional and sweeping methods was compared using a t-test, and the degree of bleeding and damage to the mucosa was compared using Fisher’s exact test. The sensitivity, specificity, accuracy, positive predictive value (PPV), and negative predictive value (NPV) were calculated, including the 95% confidence intervals (CI). Cohen’s kappa coefficient was used to determine the agreement between the sweeping method and either the conventional method, the histopathology, or PCR. Kappa values can be interpreted as follows: < 0.00, poor; 0.00–0.20, slight; 0.21–0.40, fair; 0.41–0.60, moderate; 0.61–0.80, substantial; 0.81–1.00, almost perfect^30^. The receiver operating characteristic curve was calculated, with the corresponding 95% CI, and the statistical difference in the area under the receiver operating characteristic (AUROC) between the two methods was evaluated using the method of DeLong et al.^31^ All reported *P*-values were two-sided, and a *P*-value of < 0.05 was considered significant. Analyses were performed using SAS version 9.4 (SAS Institute Inc., Cary, NC, USA).

## Supplementary information

Supplementary Information 1.

Supplementary Video 1.

## Data Availability

The datasets analyzed during the current study are available from the corresponding author on reasonable request.
