# Supplementary material for: Diagnostic accuracy of “sweeping” method compared to conventional sampling in rapid urease test for Helicobacter pylori detection in atrophic mucosa
Source: Sci Rep. 2020 Oct 28;10:18483. doi: 10.1038/s41598-020-75528-1 (PMC7595103; doi:10.1038/s41598-020-75528-1)
Supplement: Supplementary file 1 — Supplementary Information 1. [file 41598_2020_75528_MOESM1_ESM.pdf]

Diagnostic accuracy of "sweeping" method compared to conventional sampling in rapid urease test for *Helicobacter pylori* detection in atrophic mucosa

Choong-Kyun Noh, Gil Ho Lee, Jin Woong Park, Jin Roh, Jae Ho Han, Eunyoung Lee, Bumhee Park, Sun Gyo Lim, Sung Jae Shin, Jae Youn Cheong, Jin Hong Kim, and Kee Myung Lee*

Supplementary Figures

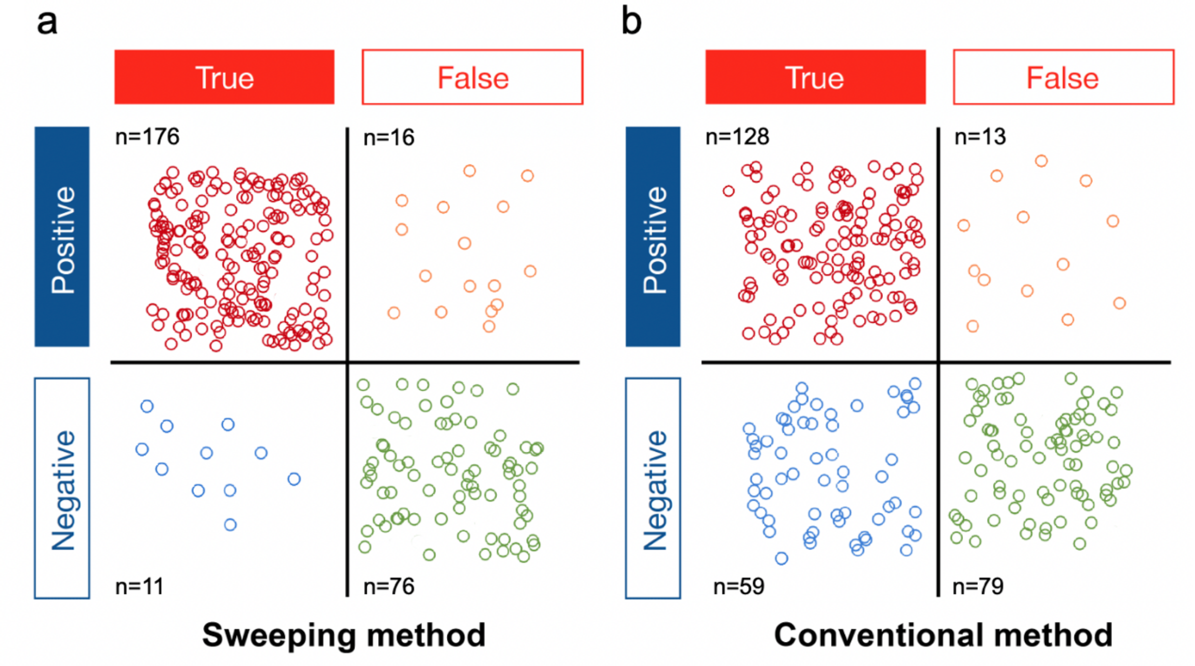

Supplementary Figure 1. Frequency distribution according to the *Helicobacter pylori* detection status (each circle was randomly distributed).

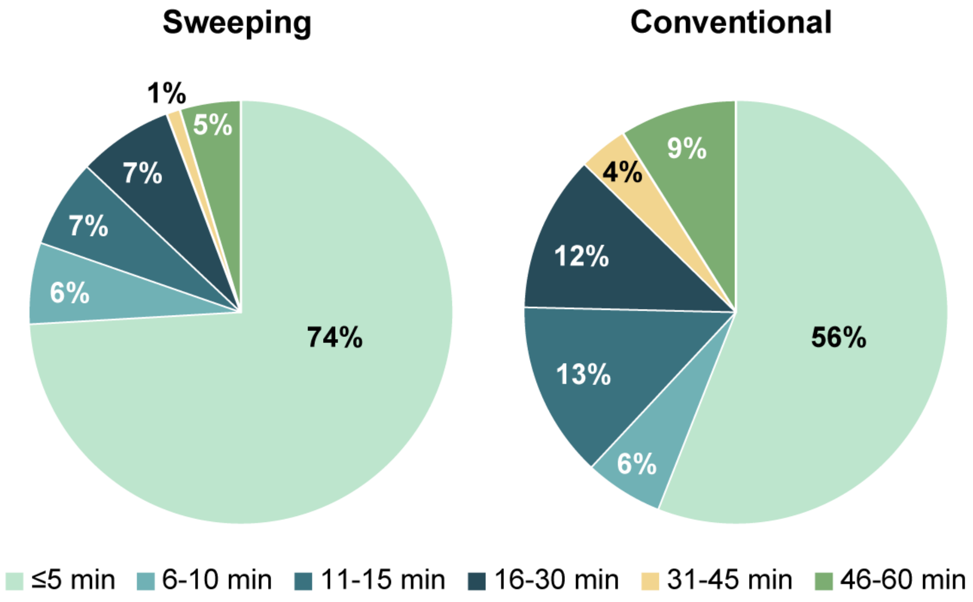

Supplementary Figure 2. Comparison of the distribution of detection time between the sweeping and the conventional method.

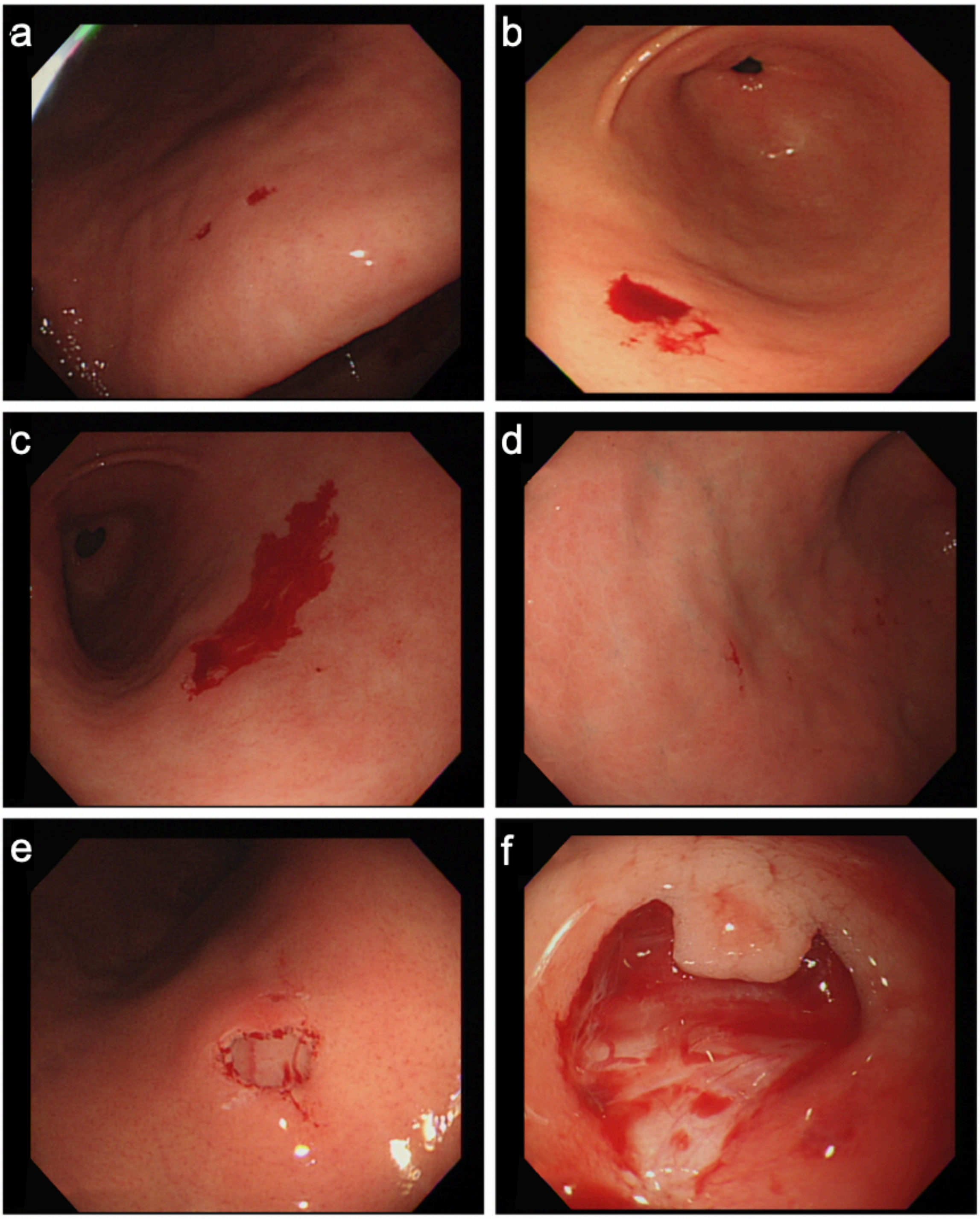

Supplementary Figure 3. Damage to the stomach mucosa that may occur during forceps biopsy, showing the extent of damage possible as follows: no bleeding (**a**), minimal bleeding (**b**), oozing blood (**c**), no mucosal damage (d), submucosal layer exposure (**e**), and muscle exposure (**f**).

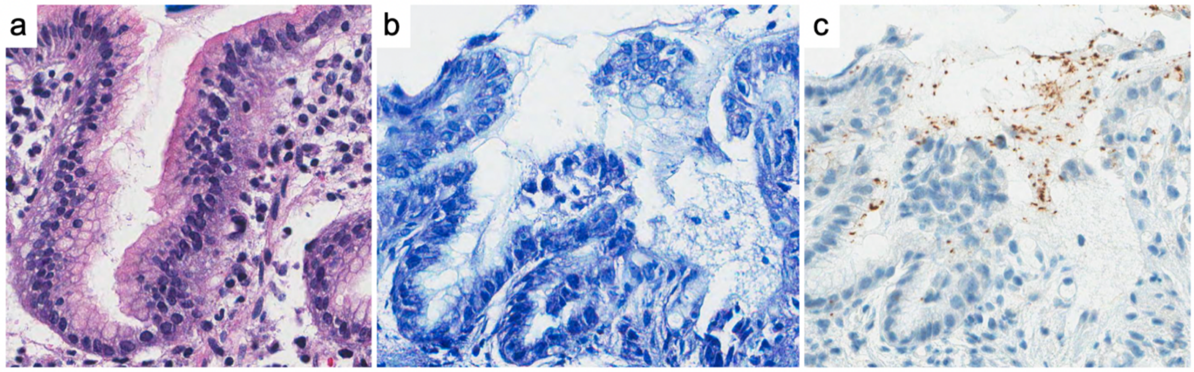

Supplementary Figure 4. Results of the histopathology-based detection of *Helicobacter pylori*, showing the hematoxylin and eosin staining, ×400 (**a**), Giemsa staining, ×400 (**b**), and immunohistochemical staining, ×400 (**c**).

Supplementary Table 1. Comparison of adverse events for the sweeping and the conventional method

Adverse events	Sweeping	Conventional	*P*-value
Bleeding, n (%)			< 0.001
No bleeding	271 (97.1)	37 (13.3)	
Minimal	5 (1.8)	210 (75.3)	
Oozing	3 (1.1)	30 (10.8)	
Spurting		2 (0.7)	
Damage, n (%)			< 0.001
No	273 (97.9)		
Superficial	6 (2.2)	235 (84.2)	
Submucosa exposure		33 (11.8)	
Muscle exposure		11 (3.9)	
